# EMBRACING OPPORTUNITIES TO ENGAGE IN WORK ON POLICIES AND PROGRAMS ON ELDER RIGHTS

**DOI:** 10.1093/geroni/igae098.0197

**Published:** 2024-12-31

**Authors:** Pamela Teaster

**Affiliations:** Virginia Tech., BLACKSBURG, Virginia, United States

## Abstract

The Health and Aging Policy Fellowship (HAPF) provides fellows with an unprecedented opportunity to understand, to experience, and to delve into various dimensions of policy making with the goal of improving the health and well-being of present and future older adults and their families. The purpose of this presentation is to discuss opportunities that my fellowship placement afforded to work on elder rights policies with staff of the Administration for Community Living. First, I will provide an overview of ACL activities and responsibilities. Then, specifically, I will discuss policy making challenges and opportunities related to the projects on which I worked: Adult Protective Services, the Long-Term Care Ombudsman Program, and guardianship. I will conclude the presentation with how the experiences I had with the HAPF have shaped my present and future research, instruction, and outreach.

